# Avoidance behaviors and negative psychological responses in the general population in the initial stage of the H1N1 pandemic in Hong Kong

**DOI:** 10.1186/1471-2334-10-139

**Published:** 2010-05-28

**Authors:** Joseph TF Lau, Sian Griffiths, Kai Chow Choi, Hi Yi Tsui

**Affiliations:** 1 Centre for Health Behaviours Research, School of Public Health and Primary Care, The Chinese University of Hong Kong, Hong Kong SAR, China; 2 Centre for Medical Anthropology and Behavioral Health, Sun Yat-Sen University, Guangzhou, China; 3 School of Public Health and Primary Care, The Chinese University of Hong Kong, Hong Kong SAR, China

## Abstract

**Background:**

During the SARS pandemic in Hong Kong, panic and worry were prevalent in the community and the general public avoided staying in public areas. Such avoidance behaviors could greatly impact daily routines of the community and the local economy. This study examined the prevalence of the avoidance behaviors (i.e. avoiding going out, visiting crowded places and visiting hospitals) and negative psychological responses of the general population in Hong Kong at the initial stage of the H1N1 epidemic.

**Methods:**

A sample of 999 respondents was recruited in a population-based survey. Using random telephone numbers, respondents completed a structured questionnaire by telephone interviews at the 'pre-community spread phase' of the H1N1 epidemic in Hong Kong.

**Results:**

This study found that 76.5% of the respondents currently avoided going out or visiting crowded places or hospitals, whilst 15% felt much worried about contracting H1N1 and 6% showed signs of emotional distress. Females, older respondents, those having unconfirmed beliefs about modes of transmissions, and those feeling worried and emotionally distressed due to H1N1 outbreak were more likely than others to adopt some avoidance behaviors. Those who perceived high severity and susceptibility of getting H1N1 and doubted the adequacy of governmental preparedness were more likely than others to feel emotionally distressed.

**Conclusions:**

The prevalence of avoidance behaviors was very high. Cognitions, including unconfirmed beliefs about modes of transmission, perceived severity and susceptibility were associated with some of the avoidance behaviors and emotional distress variables. Public health education should therefore provide clear messages to rectify relevant perceptions.

## Background

The WHO raised the influenza alert level to the highest pandemic 'Phase 6' level on June 11, 2009. As of June 19, 2009, 44,287 confirmed H1N1 cases were detected in 88 countries, territories and areas and 180 deaths had been reported [1]; the number of death increased to 15,292 as of February 7, 2010. A preliminary study showed that the new H1N1 virus is more infectious than seasonal influenza [2]. In Hong Kong, the first confirmed case, a traveler from Mexico, was reported on May 1, 2009, leading to the closure and isolation of the Metropark Hotel and to the quarantining of 350 guests and staff from May l to May 8, 2009. During the H1N1 outbreak, the Hong Kong Hospital Authority raised the alert level to the highest 'Emergency Response Level'. The government maintained its confinement strategy till 12 June, 2009, when it became obvious that cases were spread in the community, and the response thus became mitigation. As of June 27, 2009, there are 629 confirmed cases in Hong Kong and no H1N1-related death was recorded and as of February 10, 2010, there were 67 H1N1-related deaths.

The lessons learned from the SARS experience in Hong Kong [3] and other countries demonstrated the importance of understanding community responses [4,5]. Surveillance of community responses at the initial phase of an emerging epidemic is useful to inform both policy makers and the public about the state of preparedness. In Hong Kong, at the time of the SARS epidemic, the perceptions and behaviors changed dramatically during the course of the outbreak [4,6-8]. Panic and worry increased and became widespread during the epidemic and remained high in the post-SARS period [4,9]. At the height of the epidemic, the general public avoided going out, traveling to other countries and gathering for social activities [4]. Scholars estimated that a loss of HK$15 billion in spending on goods and services in Hong Kong domestic economy was attributable to the SARS epidemic [10].

Similar studies were conducted to investigate community responsiveness to the threat of human-to-human H5N1 avian flu transmissions in Hong Kong [11-14]. Previous studies on human avian flu or SARS in different countries also suggested that widespread distress occurred in affected areas and nationwide populations even at the early phase of the outbreak, causing serious social and economic disruption [15,16].

A study was conducted to investigate community behavioral and emotional responses at the very initial phase after the identification of the first few H1N1 cases in Hong Kong [17]. A few other studies have investigated community's attitudinal and behavioral responses toward the early phase of the H1N1 pandemic in countries including the U. K. [18], Australia [19], Malaysia and Europe [20], France [21], Japan [22]. Avoidance behaviors have been prevalent in a number of countries or cities, such as Hong Kong and Malaysia but not in the U. K. Mild emotional distress was observed in Hong Kong but the public in Japan perceived overwhelming fear. The majority of the respondents in the Hong Kong study washed their hands more often than usual, but only around 30% of those in the U. K. did the same. Variations in perceived susceptibility and perceived efficacy over preventive measures have also been reported in these studies. Therefore, community responses to the H1N1 pandemic are likely to be country-specific, possibly determined by previous experiences of epidemics such as SARS, the health system, risk communication patterns and even culture [22].

This study investigated whether the general population in Hong Kong avoided visiting different places (going out, visiting crowded places and visiting hospitals) and assessed some negative psychological responses to H1N1, including whether people were much worried about contracting H1N1 and their level of emotional distress (panicking, depression or emotional disturbance) due to H1N1. Factors in association with the outcome variables on avoidance behaviors and negative psychological responses were investigated, including variables such as socio-demographic characteristics, confirmed knowledge and unconfirmed beliefs about modes of H1N1 transmission, evaluation towards governmental preparedness/performance, perceived availability of treatment, and risk perception (perceived severity and susceptibility related to H1N1). The study period of this report covers almost the entire pre-pandemic and pre-community outbreak phase of the H1N1 epidemic in Hong Kong.

## Methods

### Sampling and data collection

The study population comprised all Chinese adults who were 18 years old or above in Hong Kong. Anonymous telephone interviews were conducted by well-trained interviewers, using an identical structured questionnaire, from May 7 to May 9 (Day 7-9, n = 550), from May 14 to May 17 (Day 14-17, n = 201), and from June 4 to June 6 (Day 34-36, n = 248), 2009. There were respectively 1, 2 and 30 imported cases (and no community non-imported cases) detected at the beginning date of these 3 surveys. Preliminary data from the survey conducted from May 7 to May 9 have previously been reported [17]. The first local community-infected case with an unknown source of infection was reported on June 11, 2009, so that the surveys (May 7 to June 6, 2009) therefore covered almost the entire 'pre-community outbreak phase' (May 1 to June 10, 2009) of the local epidemic. Random telephone numbers were selected from an up-to-date telephone directory and the last two digits of the selected telephone number were randomized to include some unlisted telephone numbers; over 95% of the households in Hong Kong have a fix-line telephone at home [23]. The interviews were conducted from 6:30 to 10 pm to avoid over-representing the non-working population. One member was selected by the last-birthday-rule from each of the contacted households. At least 3 phone calls were made at different hours and days before an unanswered number is considered invalid. Verbal consent was sought and the study was approved by the ethics committee of the Chinese University of Hong Kong.

A total of 2,583 phone numbers were made and being answered by someone (2,906 calls were unanswered with at least 3 attempts made), out of which 1,621 were eligible households were identified and being invited to join the study. Of these 1,621 eligible respondents, 95 (5.9%) could not be contacted after 3 attempts, 525 (32.4%) refused to join or withdrew from the study, and 999 (61.6%) participated in the study. Previously, preliminary data from the May 7 to May 9 survey have been reported elsewhere [17].

### Measures

Dependent variables included current avoidance behaviors: 1) 'avoided going out', 2) 'avoided visiting crowded places' and 3) 'avoided visiting hospitals', and exhibition of negative psychological responses: 1) worried very much that oneself or one's family would contract H1N1 and 2) emotional distress ('panicking very much' or 'felt much depressed' or 'felt much emotionally disturbed' due to H1N1).

Socio-demographic characteristics were recorded. Correct knowledge and unconfirmed beliefs about modes of H1N1 transmission were assessed. Respondents were asked about perceived availability of treatment. Risk perception questions include those related to perceived severity of H1N1 (fatality and severe irreversible bodily damages), the relative chance for Hong Kong to have a large-scale H1N1 outbreak as compared to other countries, and perceived susceptibility (oneself, one' family and the general public). Questions were also asked to evaluate relevant actions taken by the government (6 items), their ability to control the epidemic (2 items), as well as the health system's preparedness toward the H1N1 pandemic (3 items: adequacy of medicine, vaccines and personal protection equipments). These items are listed in Tables 1, 2 and 3. They were modified from the questionnaires which had been used in some avian flu studies [11-14] and SARS studies [4,9,24,25]. They have also been used in the published baseline H1N1 study [17].

**Table 1:** Background characteristics of the respondents (n = 999).

	**%**^**#**^
**Socio - demographic characteristics**	
Gender	
Male	43.4%
Female	56.6%
Age	
<30	25.0%
30 - 39	20.1%
40 - 49	27.1%
50 - 60	27.7%
Education level	
Form 3 or below	18.5%
Form 4 - matriculation	46.4%
College or above	35.1%
Marital status	
Single	33.5%
Married/cohabited	65.0%
Divorced/widowed	1.5%
Full-time employed	
No	44.0%
Yes	56.0%
Being health care workers	
No	98.1%
Yes	1.9%

*Basing on end-2008 Census data (Census & Statistics Department HKSAR, 2009), the proportions of male and female of age 18-60 in Hong Kong were respectively 46.02% and 53.98%; the proportions of the 4 age groups in Hong Kong (<30, 30-39, 40-49, and 50-60) were respectively 24.75%, 23.78%, 27.33%, and 24.14%.

^#^Less than 0.5% missing cases exist for the listed variables.

**Table 2:** Perceptions related to H1N1 (n = 999).

	**%**^**#**^
**Unconfirmed beliefs and knowledge about modes of transmission of H1N1**	
***Unconfirmed beliefs about modes of transmission***	
Airborne with a long distance (from one building to another one)	36.7%
Transmitted via water sources (e.g. reservoirs)	35.7%
Transmitted via insect bites	22.9%
Transmitted via well-cooked pork	41.6%
*Any one of above*	*61.8%*
	
***Correct knowledge about modes of transmission***	
Could be transmitted via droplets (e.g. sneeze)	97.5%
Could be transmitted via touching body of infected persons	74.2%
Could be transmitted via touching contaminated objects	77.8%
*All above items being correct*	*60.3%*
	
**Evaluation of governmental preparedness and performance in dealing with H1N1**	
***Preparedness***	
The local health system do not have enough medication for treating H1N1	36.2%
The local health system do not have enough vaccine for preventing H1N1	41.1%
Hospitals in Hong Kong do not have enough personal protection equipments for preventing H1N1	30.2%
*Any one of the above*	*52.6%*
	
***Perceived ability to control the epidemic***	
Hong Kong will be able to control the H1N1 epidemic (agree)	83.6%
Hong Kong government will be certainly/most likely/likely able to control a large-scale H1N1 outbreak	79.5%
*Any one of the above*	*92.3%*
	
Evaluation of governmental performance *	
Overall average evaluation score	
≤5	10.7%
>5 - 8	69.1%
>8	20.2%
**Perceived availability of treatment**	
There is no effective drug for the treatment of H1N1	38.9%
	
**Risk perception**	
***Perceived severity of H1N1***	
High fatality	20.6%
Severe irreversible body damages	18.9%
	
***Perceived chance of having a large scale H1N1 outbreak in Hong Kong in the future year, compared to other countries***	
Hong Kong = other countries	45.2%
Hong Kong > other countries	7.1%
Hong Kong < other countries	47.6%
	
***Perceived high or very high chance of contracting H1N1 in the coming year***	
The respondent	8.6%
Family members	8.7%
The general public	12.5%

* Governmental performance was assessed by 6 items: Timeliness of measures taken; effectiveness of implemented measures; clear explanations made to citizens; adequacy of Implemented measures; coordination across governmental departments; overall performance of the government. (Score range = 0 to 10, with 5 as the passing mark). An average was calculated for the 6 item scores.

^#^Less than 2% missing cases exist for the listed variables.

**Table 3:** Prevalence of avoidance behaviors and negative psychological responses (n = 999).

	**%**^**#**^
**Avoidance behaviors**	
Avoid going to crowed places	54.9%
Avoid going out unless necessary	44.0%
Avoid going to hospitals	63.4%
*Any of the above*	*76.5%*
	
**Negative psychological responses**	
***Worry for contracting H1N1***	
Respondent worrying himself/herself would contract the disease	11.7%
Respondent worrying his/her family members would contract the disease	15.3%
*Any of the above*	*15.8%*
	
***Emotional distress***	
Feeling much in panic	4.4%
Feeling much depressed	2.2%
Feeling much emotionally disturbed	3.4%
*Any of the above*	*6.0%*

^#^Less than 2% missing cases exist for the listed variables.

### Data analysis

Associations between the independent variables and the dependent variables (avoidance behaviors and negative psychological responses) were assessed by using univariate odds ratios (OR) and their respective 95% confidence intervals (CI). Variables that were significant in the univariate analysis were used as candidates for fitting logistic regression models. Multivariate OR and their 95% CI were reported. SPSS 16.0 was used for the data analyses with p < .05 as the level of statistical significance.

## Results

### Background Characteristics

Of all respondents (n = 999), 43.4% were males; 54.8% were of age 40 years old or above; 35.1% received some post-secondary education; 65% were currently married or were cohabitating with someone; 56% were currently employed full time; and 1.9% were health care workers. The age and gender distributions did not vary across the 3 surveys and were similar to those of the census data (footnote of Table 1).

### Perceptions Related to H1N1

#### Unconfirmed beliefs and correct knowledge about modes of transmission of H1N1

Of all respondents, 61.8% held at least one of the unconfirmed beliefs that H1N1 could be transmitted through airborne spread across long distance (e.g. from a building to another building; 36.7%), via water sources such as reservoirs (35.7%), via insect bites (22.9%) or via eating well cooked pork (41.6%). Respectively, 97.5%, 74.2% and 77.8% of the respondents correctly knew that H1N1 is transmittable via droplets, touching the body of infected person or contaminated objects; about 60.3% were correct in all these three items (Table 2).

#### Evaluation of governmental preparedness and performance in dealing with H1N1

Around 30-40% of all respondents believed that the local health system currently did not have enough medication (36.2%), vaccine (41.1%) or personal protection equipments (30.2%) to deal with the H1N1 epidemic, with 52.6% holding at least one of such 3 beliefs (Table 2). The majority (92.3%) of the respondents was confident in the public's or governmental ability to control the epidemic, i.e., either agreeing with the statements 'Hong Kong would be able to control the H1N1 epidemic' (83.6%) or with the statement that the 'Hong Kong government would be able to control a large-scale H1N1 outbreak' (79.5%). The majority (89.3%) of the respondents gave a passing score >5 for the governmental performance in dealing with H1N1 (range = 0 to 10, with 5 as the passing mark; Table 2).

#### Perceived availability of treatment

About 39% (38.9%) of the respondents believed that there was so far no effective drug available to treat H1N1 (Table 2).

#### Risk perceptions

Around 20% of the respondents believed that H1N1 is highly fatal (20.6%) or could cause severe irreversible bodily damages (18.9%; Table 2). Respectively, 7.1% and 47.6% believed that Hong Kong has a higher or a lower chance of having a large scale H1N1 outbreak in the future year, as compared to other countries. Close to 10% of the respondents perceived a high or very high chance for himself/herself (8.6%), his/her family members (8.7%) or the general public (12.5%) to contract H1N1 in the next year (Table 2).

### Prevalence of Avoidance Behaviors and Negative Psychological Responses

Respectively 54.9%, 44.0% and 63.4% of the respondents currently avoided going to crowded places, avoided going out or avoided visiting hospitals. Around 15% (15.8%) of the respondents were currently much worried that either they or their family members would contract H1N1; 6.0% showed signs of emotional distress (i.e. panicking very much or felt much depressed or were very much emotionally disturbed due to H1N1).

### Factors Associated with Avoidance Behaviors

Females, older respondents, those with >= 1 unconfirmed beliefs about modes of H1N1 transmission, those who knew that H1N1 could be transmitted 'via droplets', 'bodily contact with infected person' or 'touching contaminated objects', those who were very worried that either they or their family members would contract H1N1, those expressing emotional distress (in panic or feeling very depressed or being highly emotionally disturbed due to H1N1) were more likely than others to avoid visiting crowded places (multivariate OR = 1.42 to 3.90, p < .05; Table 4).

**Table 4:** Factors associated with avoidance behaviors.

	Avoided visiting crowded places			Avoided going out			Avoided visiting hospitals		
			
	Row %	**OR**_**U**_	**OR**_**m **_**(95% CI)**	Row %	**OR**_**U**_	**OR**_**m **_**(95% CI)**	Row %	**OR**_**U**_	**OR**_**m **_**(95% CI)**
**Wave of survey**									
Wave 1	57.5	1.00	NS	47.8	1.00	NS	67.2	1.00	NS
Wave 2	56.5	0.96		43.5	0.84		57.7	0.67*	
Wave 3	48.0	0.68*		35.9	0.61**		59.7	0.72*	
**Background characteristics**									
Gender									
Male	48.5	1.00	1	37.4	1.00	1	59.2	1.00	NS
Female	59.8	1.58***	1.51 (1.15 - 1.98)**	49.0	1.61***	1.42 (1.07 - 1.89)*	66.7	1.38*	
Age									
<30	38.0	1.00	1	28.8	1.00	1	-	-	-
30 - 39	53.7	1.89**	1.81 (1.22 - 2.69)**	44.8	2.00***	2.17 (1.41 - 3.33)***	-	-	
40 - 49	55.9	2.07***	2.14 (1.48 - 3.10)***	42.8	1.85**	2.09 (1.41 - 3.11)***	-	-	
50 - 60	70.0	3.81***	3.90 (2.67 - 5.70)***	58.3	3.46***	3.66 (2.49 - 5.38)***	-	-	
Education level									
Form 3 or below	69.6	1.00	NS	57.6	1.00	NS	-	-	-
Form 4 - matriculation	54.5	0.53**		44.8	0.60**		-	-	
College or above	47.7	0.40***		35.7	0.41***		-	-	
Marital status									
Single	40.8	1.00	NS	31.3	1.00	NS	56.8	1.00	1
Married/cohabited	62.2	2.39***		50.5	2.24***		67.2	1.56**	1.47 (1.11 - 1.95)**
Divorced/widowed	46.7	1.27		33.3	1.10		53.3	0.87	0.79 (0.28 - 2.29)
Full-time employed									
No	-	-	-	50.2	1.00	1	-	-	-
Yes	-	-		38.8	0.63***	0.72 (0.53 - 0.97)*	-	-	
**Unconfirmed beliefs and knowledge about modes of transmission of H1N1**									
Unconfirmed beliefs about modes of transmission ^†^									
None	46.8	1.00	1	34.5	1.00	1	55.8	1.00	1
At least one item	60.0	1.70***	1.67 (1.27 - 2.21)***	49.9	1.89***	1.88 (1.41 - 2.51)***	68.2	1.70***	1.56 (1.18 - 2.05)**
Correct knowledge about modes of transmission ^‡^									
Not all items being correct	48.2	1.00	1	37.9	1.00	1	59.2	**1.00**	NS
All items being correct	59.2	1.56**	1.42 (1.08 - 1.87)*	47.9	1.51**	1.43 (1.08 - 1.88)*	66.3	1.35*	
**Evaluation of governmental preparedness and performance in dealing with H1N1**									
Inadequacy of government preparation (health system) ^§^									
None	-	-	-	40.5	1.00	NS	-	-	-
At least one of the three items	-	-		47.3	1.32*		-	-	
Perceived governmental ability of controlling the epidemic ^¶^									
None	-	-	-	29.9	1.00	NS	-	-	-
At least one of the two items	-	-		45.2	1.94*		-	-	
Governmental performance in dealing with H1N1 (average score of 6 items) ^#^									
≤5	49.5	1.00	NS	37.1	1.00	NS	58.1	1.00	NS
>5 - 8	53.0	1.15		42.8	1.27		62.3	1.19	
>8	64.1	1.82*		51.0	1.76*		69.5	1.65*	
Risk perception									
Perceived severity of H1N1									
High fatality									
Disagree/unsure	52.4	**1.00**	**NS**	41.8	**1.00**	**NS**	61.6	**1.00**	**NS**
Agree	64.4	1.64**		52.4	1.53**		70.4	1.48*	
Severe irreversible bodily damages									
Disagree/unsure	52.8	**1.00**	**NS**	40.9	**1.00**	**1**	60.6	**1.00**	**1**
Agree	63.8	1.58**		57.1	1.93***	1.54 (1.09 - 2.18)*	75.7	2.02***	1.81 (1.24 - 2.63)**
Perceived chance of having a large scale H1N1 outbreak in Hong Kong in the future year, compared to other countries									
Hong Kong = other countries	-	**-**	**-**	40.4	**1**	**NS**	-	**-**	**-**
Hong Kong > other countries	-	**-**		44.3	**1.18**		-	**-**	
Hong Kong < other countries	-	**-**		47.2	1.32*		-	**-**	
									
Negative psychological responses									
Worry much that oneself or family member would contract the disease									
No	52.1	**1.00**	**1**	41.5	**1.00**	**NS**	61.0	**1.00**	**1**
Yes	69.6	2.10***	1.62 (1.07 - 2.47)*	57.5	1.89***		76.6	2.10***	1.74 (1.16 - 2.61)**
Emotional distress (Feeling much in panic or much depressed or much emotionally disturbed)									
No	53.0	**1.00**	**1**	42.4	**1.00**	**1**	62.5	**1.00**	**NS**
Yes	81.7	3.94***	3.04 (1.42 - 6.48)**	66.1	2.65**	2.61 (1.42 - 4.79)**	76.7	1.97*	

*p < 0.05; **p < 0.01; ***p < 0.001; OR_U_: univariate odds ratio obtained using logistic regression; OR_m_: odds ratio obtained from stepwise multivariate logistics regression analysis using univariately significant variables as candidate variables; NS: not statistically significant in multivariate analysis.

^† ^Unconfirmed beliefs about modes of transmission was assessed by 4 items: the disease could be airborne across a long distance (e.g. from one building to another one); transmitted via water sources (e.g. reservoirs); transmitted via insect bites; transmitted via well-cooked pork.

^‡ ^Correct knowledge about modes of transmission was assessed by 3 items: the disease could be transmitted via droplets (e.g. sneeze); could be transmitted via touching body of infected person; could be transmitted via touching contaminated objects.

^§ ^Inadequacy of government preparation (health system) was assessed by 3 items: local health system do not have enough medication for treating H1N1; local health system do not have enough vaccine for preventing H1N1; hospitals in Hong Kong do not have enough personal protection equipments for preventing H1N1.

^¶ ^Perceived governmental ability of controlling the epidemic was assessed by 2 items: Hong Kong will be able to control the H1N1 epidemic; Hong Kong government is able to control a large-scale H1N1 outbreak.

^# ^Governmental performance was assessed by 6 items: Timeliness of measures; effectiveness of implemented measures; clear explanations made to citizens; adequacy of implemented measures; coordination across governmental departments; overall performance of the government. (Score range = 0 to 10, with 5 as the passing mark). An average score was calculated for the 6 item scores.

Variables that were not significantly associated with any of the dependent variables in the univariate analysis were not tabulated. These variables include being current health care practitioner, perceived availability of drugs, perceived high chances of contracting the disease for himself/herself, his/her family members and the general public.

Females, older respondents, those with >= 1 unconfirmed beliefs about modes of H1N1 transmission, those who knew that H1N1 could be transmitted 'via droplets', 'bodily contact with infected person' or 'touching contaminated objects', those who believed that H1N1 would cause severe irreversible bodily damage, and those expressing emotional distress (in panic or feeling very depressed or being highly emotionally disturbed due to H1N1) were more likely than others to avoid going out (multivariate OR = 1.42 to 3.66, p < .05). Those who were full-time employed were less likely than others to avoid going out (multivariate OR = 0.72, p < .05; Table 4).

Respondents who were married/cohabited, those with >= 1 unconfirmed beliefs about modes of H1N1 transmission, those who believed that H1N1 would cause severe irreversible bodily damage, and those who were very worried that either they or their family members would contract H1N1 were more likely than others to avoid visiting hospitals (multivariate OR = 1.47 to 1.81, p < .05; Table 4).

### Factors Associated with Negative Psychological Responses

The results of the multivariate analysis showed that those who believed H1N1 would cause severe irreversible bodily damage and those who believed that they themselves had a high chance of contracting H1N1 were more likely than others to be much worried that either they or their family members would contract H1N1 (multivariate OR = 1.95 and 3.31 respectively, p < .05; Table 5). Those who believed either that the general public and/or the local government would be able to control a large scale local H1N1 outbreak were less likely to show the worry (multivariate OR = 0.51).

**Table 5:** Factors associated with negative psychological responses.

	Feeling much worried either oneself or one's family members would contract H1N1			Expressed emotional distress (being much in panic or felt very much depressed or being highly emotionally disturbed)		
		
	Row %	**OR**_**U**_	**OR**_**m **_**(95% CI)**	Row %	**OR**_**U**_	**OR**_**m **_**(95% CI)**
**Background characteristics**						
Gender						
Male	13.4	1.00	-	3.7	1.00	1
Female	17.7	1.39		7.8	2.21**	2.17 (1.18 - 4.00)*
Education level						
Form 3 or below	-	-	-	9.2	1.00	NS
Form 4 - matriculation				5.9	0.61	
College or above				4.6	0.47*	
Full-time employed						
No	-	-	-	7.8	1.00	NS
Yes				4.7	0.58*	
						
**Unconfirmed beliefs and knowledge about modes of transmission of H1N1**						
Unconfirmed beliefs about modes of transmission ^†^						
None	12.3	1.00	NS	-	-	-
At least 1 item	18.0	1.56*				
						
**Evaluation of governmental preparedness and performance in dealing with H1N1**						
Inadequacy of government preparation (health system) ^‡^						
None	-	-	-	3.6	1.00	1
At least 1 of 3 items				8.1	2.34**	2.35 (1.30 - 4.26)**
Perceived governmental ability in controlling the epidemic ^§^						
None	26.0	1.00	**1**	-	-	-
At least 1 of 2 items	14.9	0.50*	0.51 (0.29 - 0.90)*			
						
**Risk perception**						
***Perceived severity of H1N1***						
High fatality						
Disagree/unsure	14.4	1.00	NS	5.1	1.00	1
Agree	20.9	1.57*		9.3	1.93*	1.94 (1.08 - 3.49)*
Severe irreversible bodily damages						
Disagree/unsure	13.8	1.00	1	5.4	1.00	-
Agree	24.3	2.00***	1.95 (1.30 - 2.90)**	8.5	1.62	
						
***Perceived chance of contracting H1N1 in the coming year***						
The respondent						
Low/very low/unsure	13.8	1.00	1	5.5	1.00	NS
High or very high	37.2	3.70***	3.31 (2.02 - 5.42)***	11.6	2.26*	
Family members						
Low/very low/unsure	13.8	1.00	NS	5.1	1.00	1
High or very high	36.8	3.62***		16.1	3.59***	3.68 (1.86 - 7.29)***
The general public						
Low/very low/unsure	13.7	1.00	NS	5.1	1.00	NS
High or very high	30.6	2.79***		12.1	2.58**	

*p < 0.05; **p < 0.01; ***p < 0.001; OR_U_: univariate odds ratio obtained from logistic regression models; OR_m_: odds ratios obtained from stepwise multivariate logistics regression analysis, using univariately significant variables as candidate variables; NS: not statistically significant in multivariate analysis.

^† ^Unconfirmed beliefs about modes of transmission were assessed by 4 items: the disease could be airborne across a long distance (e.g. from one building to another one); transmitted via water sources (e.g. reservoirs); transmitted via insect bites; transmitted via well-cooked pork.

^‡^Inadequacy of government preparation (health system) was assessed by 3 items: local health system do not have enough medication for treating H1N1; local health system do not have enough vaccine for H1N1; Hospitals in Hong Kong do not have enough personal protection equipments for H1N1.

^§ ^Perceived governmental ability of controlling the epidemic was assessed by 2 items: Hong Kong will be able to control the H1N1 epidemic; Hong Kong government will be able to control a large-scale H1N1 outbreak.

Variables that were not significantly associated with any of the dependent variables in the univariate analysis were not tabulated. These variables include wave of survey, age, marital status, current health care practitioner, correct knowledge about modes of transmission, perceived chance of having an H1N1 pandemic in Hong Kong as compared to other counties in the future year.

In the multivariate analysis, females, those who doubted about adequacy of governmental preparedness (inadequate vaccine or medication or personal protection equipments in Hong Kong), those who associated H1N1 with a high fatality, and those who believed that their family members had a high chance of contracting H1N1 were more likely than others to indicate emotional distress (panicking or much depressed or much emotionally disturbed) due to H1N1. The significant multivariate OR ranged from 1.94 to 3.68 (p < .05; Table 5).

## Discussion

Around 77% of the respondents showed some avoidance behaviors. The studies covered the entire early 'pre-community outbreak phase' of the H1N1 epidemic in Hong Kong during which all confirmed cases were imported. During the study period, the local government had not given any public health advice about avoiding going to different places, though a previous analysis of our May 7 to May 9 data showed that 31.6% of the public misconceived that such an advice was given [17]. Avoidance of visiting hospital may be due to the fear of getting infected in hospitals, which was prominent during the SARS period [9]. The government only started advising people to avoid crowded places at the 'community outbreak phase' of the epidemic. There seemed to be no serious immediate public health threat for going out or visiting different places. Such avoidance behaviors were associated with negative psychological responses; emotional elements may therefore be strongly involved in making the decisions. Experience from SARS showed that such avoidance behaviors among large numbers in the population potentially damages the economy and disrupts daily lives.

About half of the respondents believed that Hong Kong has a lower chance of having an H1N1 outbreak as compared to other countries, whilst only less than 10% held the opposite belief. There were signs of underestimating the risk of having a community outbreak in Hong Kong [17]. The shift into the pandemic phase as announced by the WHO and the explosion of non-imported community cases in Hong Kong (629 as of June 27, 2009) may change the picture completely. The direction of change is however uncertain. A few international studies also documented strong levels of anticipated anxiety and avoidance behaviors at the early phase of human avian flu outbreaks or pandemic influenza [15,26-28]. The impact of pandemics and unknown emerging infections has not been widely studied. Avoidance behaviors and emotional distress may have been under-emphasized in the preparedness plans.

It is seen that females, older people and those who were not full-time employed were more likely than others to show avoidance behaviors or signs of emotional distress. The results are consistent with those reported during the SARS period [9]. A recent study exploring people's emotional and behavioral responses to an avian flu outbreak also showed that females and older people were, respectively, more likely to express negative emotional responses and exhibit avoidance behaviors (e.g., avoiding leaving their residence, avoiding crowds and avoiding visiting hospitals) in response to avian flu [26]. Attention should therefore be given to avoidance behaviors and psychological needs of these subpopulations at times of a pandemic.

Perceptions still count in this context. There were substantial unconfirmed beliefs about the mode of H1N1 transmission (61.8% had at least one unconfirmed belief). Around 1/4 of the respondents did not know that the virus could be spread by touching contaminated objects. The aforementioned unconfirmed beliefs about transmission mode were significantly associated with avoidance behaviors. Unconfirmed beliefs about modes of transmission were also documented in H5N1 studies [12], suggesting that similar unconfirmed beliefs exist in general for emerging respiratory infectious diseases. Rectification of misconceptions is important - and may decrease and reduce unwarranted anxiety.

Around 20% of the respondents believed that H1N1 would result in high fatality or severe irreversible bodily damages. Such beliefs may be affected by the SARS experience. Perceived high fatality was associated with emotional distress (e.g. panic) due to H1N1 and perceived severe irreversible bodily damage was associated with 3 of the 5 outcome variables on avoidance behaviors and negative psychological responses. Up-to-date information about the clinical properties of H1N1 should be disseminated to the public in layman terms.

The actual fatality associated with H1N1, both local and international, remains low. The cost of assurance by Hong Kong government is however, high - with early summer closure of all primary schools and kindergarten and a number of secondary schools, 10 billion Hong Kong dollars being spent (1.2 billion US$) to purchase H1N1 vaccines and reorganization of the health services to accommodate escalating infection figures are not insubstantial. Tourism may be adversely affected. A substantial proportion of the public may be overestimating its fatality and physical damages. Since public understanding of risk and of these mitigation measures will help to reduce unnecessary concern and changes in lifestyle amongst the population, public education is important.

As expected, perceived personal/family susceptibility for contracting H1N1 was associated with negative psychological responses due to H1N1. The association between perceived personal/family susceptibility and avoiding going out was non-significant. The results suggest that the public did not avoid going out because of feeling susceptible. Avoidance behaviors may involve an irrational element. It is speculated that the SARS experience of avoiding going to different places [29] might have a spill-over effect.

The general public evaluated the government highly in the performance and ability to control the pandemic. They however, showed reservations about the availability of medicine and vaccine and protective equipments, possibly because H1N1 was a new disease and it was not certain whether effective medicine, vaccine and equipments were then available. The positive evaluations of governmental performance and perceived ability for Hong Kong or the government to control the H1N1 outbreak were significantly associated with the outcome variables in most of the univariate analyses. Nonetheless, most of these associations were statistically non-significant in the multivariate analysis. The associations between such variables and the outcome variables (avoidance behaviors and negative psychological responses) were hence mediated by other variables, such as worry about contracting H1N1 or perceived susceptibility. These potential mediators were multivariately associated with either the avoidance variables or the negative psychological response variables.

The study has some limitations. First, this was a cross-sectional baseline study. Second, the response rate was comparable to those of other relevant published studies but some non-responder bias may still exist [9,14,30]. Some telephone numbers are unlisted and we randomized the last two digits to cover some of the unlisted numbers. Moreover, the gender and age distributions were comparable to those of the census population data. Third, results were self-reported and social desirability bias may exist. The study was however anonymous. Fourth, Hong Kong went through unique SARS experience, the results may not be comparable with those of other countries. Fifth, the measures on negative psychological responses were based on those used in previous studies, rather than derived from some validated scales. Finally, the study was not intended to track changes within the short study period of a month - interactions between time and various independent variables were not explored. Similar data obtained from other countries are becoming available and can be compared with ours.

## Conclusion

In sum, the results of this study documented that a noticeable proportion of the public exhibited avoidance behaviors that had not been advised by the government and negative psychological responses at the early 'pre-community outbreak phase' of the H1N1 outbreak. With the relatively mild nature of the H1N1, and with all the hygiene and public health measures continually re-emphasized, an open debate on whether the public should avoid going out during the H1N1 outbreak should be encouraged. It would facilitate appropriate responses and daily lives of people in Hong Kong, one of the most densely populated cities in the world, remain undisrupted.

The study is part of an ongoing surveillance program, which is in place in Hong Kong. Hong Kong is now in the pandemic and 'community outbreak phase'. The public needs to be better informed about the modes of transmission and clinical consequences of the disease to make rational behavioral choices. Early detection of mental health problems and primary preventions are warranted. Comparisons with other parts of the world, such as mainland China, would be very informative. This study provides a better understanding of the factors associated with negative psychological responses due to H1N1, which would give useful insights to designing primary prevention of mental health distress at the initial phase of this and outbreaks of other emerging respiratory infectious diseases.

## Competing interests

The authors declare that they have no competing interests.

## Authors' contributions

JTFL designed and oversaw the study and wrote the manuscript. SG and HYT proposed suggestions to improve study and revised the manuscript intellectually. KCC performed the data analysis. All authors read and approved the manuscript.

## Disclaimer

The opinions expressed by authors contributing to this journal do not necessarily reflect the opinions of the Centre for Health Behaviours Research or the institutions with which the authors are affiliated.

## Pre-publication history

The pre-publication history for this paper can be accessed here:

http://www.biomedcentral.com/1471-2334/10/139/prepub
